# Theoretical analysis of hydrogen spillover mechanism on carbon nanotubes

**DOI:** 10.3389/fchem.2015.00002

**Published:** 2015-02-02

**Authors:** Rosalba Juarez-Mosqueda, Andreas Mavrandonakis, Agnieszka B. Kuc, Lars G. M. Pettersson, Thomas Heine

**Affiliations:** ^1^School of Engineering and Science, Jacobs University BremenBremen, Germany; ^2^Department of Physics, AlbaNova University Center, Stockholm UniversityStockholm, Sweden

**Keywords:** carbon nanotube, hydrogen spillover, hydrogen storage, DFT, Pt catalyst, circumcoronene

## Abstract

The spillover mechanism of molecular hydrogen on carbon nanotubes in the presence of catalytically active platinum clusters was critically and systematically investigated by using density-functional theory. Our simulation model includes a Pt_4_ cluster for the catalyst nanoparticle and curved and planar circumcoronene for two exemplary single-walled carbon nanotubes (CNT), the (10,10) CNT and one of large diameter, respectively. Our results show that the H_2_ molecule dissociates spontaneously on the Pt_4_ cluster. However, the dissociated H atoms have to overcome a barrier of more than 2 eV to migrate from the catalyst to the CNT, even if the Pt_4_ cluster is at full saturation with six adsorbed and dissociated hydrogen molecules. Previous investigations have shown that the mobility of hydrogen atoms on the CNT surface is hindered by a barrier. We find that instead the Pt_4_ catalyst may move along the outer surface of the CNT with activation energy of only 0.16 eV, and that this effect offers the possibility of full hydrogenation of the CNT. Thus, although we have not found a low-energy pathway to spillover onto the CNT, we suggest, based on our calculations and calculated data reported in the literature, that in the hydrogen-spillover process the observed saturation of the CNT at hydrogen background pressure occurs through mobile Pt nanoclusters, which move on the substrate more easily than the substrate-chemisorbed hydrogens, and deposit or reattach hydrogens in the process. Initial hydrogenation of the carbon substrate, however, is thermodynamically unfavoured, suggesting that defects should play a significant role.

## Introduction

Hydrogen spillover has been proposed as a promising mechanism for hydrogen storage (Lueking and Yang, 2004; Marella and Tomaselli, 2006; Li and Yang, 2006a,b; Chen and Huang, 2007, 2008; Liu et al., 2007; Zacharia et al., 2007; Zieliñski et al., 2007; Bhowmick et al., 2011). Over the past 9 years, Yang and coworkers (Lachawiec et al., 2005; Yang et al., 2006; Li and Yang, 2006a,b; Yang and Wang, 2009; Chen and Yang, 2010) have reported that hydrogen spillover is a viable technique to achieve high hydrogen storage on different carbon-based materials, even at ambient conditions. Carbon-based materials, such as carbon nanofibres (Lueking and Yang, 2004; Marella and Tomaselli, 2006), graphite (Mitchell et al., 2003a,b), and carbon nanotubes (CNTs) (Lueking and Yang, 2003, 2004; Nikitin et al., 2005, 2008; Zacharia et al., 2007; Chen and Huang, 2008; Yang and Wang, 2009; Bhowmick et al., 2011), are some of the common materials proposed as storage substrates. In 2008, Nilsson and coworkers have claimed that fully reversible hydrogenation of single-wall CNTs (SWCNT), with a diameter around 2.0 nm, can be achieved through reaction with atomic hydrogen (Nikitin et al., 2008). However, the veracity of these results and the efficiency of such graphitic materials to store hydrogen are still questionable. In two comprehensive reviews and an experimental work, Becher et al. (2003), Meregalli and Parrinello (2001) and Tibbetts et al. (2001) criticized earlier reports of hydrogen storage capacities of carbon-based materials greater than 1 wt % at ambient temperature.

A hydrogen spillover mechanism should consist of several steps: In the first, molecular hydrogen is activated and dissociated on a transition-metal catalyst (e.g., platinum, palladium, ruthenium, or nickel, Somorjai, 1994) in close contact with the substrate (e.g., carbon-based substrates). Secondly, the migration of H atoms from the catalyst particles to the substrate should occur. The last two steps consist in the diffusion and recombination of H atoms on the substrate surface (Conner and Falconer, 1995; Cheng et al., 2008; Psofogiannakis and Froudakis, 2009). Many of these steps have already been studied independently by several groups, suggesting that the picture might not be as simple as it appears. Han et al. (2012) found on the basis of quantum simulations that hydrogen migration along a graphene-like surface is an impractical process at standard conditions since it requires high-temperature heating (several 100°C). Furthermore, Chen et al. reported that, for CNTs, the curvature also has an important effect hampering the H diffusion between adjacent C atoms (Chen et al., 2007a). The authors found endothermic activation energies of 1.43, 1.07, and 0.82 eV for the H diffusion on (5,5) and (9,9) SWCNTs and graphene, respectively (Chen et al., 2007a). In addition, the migration of H atoms from a saturated Pt cluster to the carbon surface has been reported by Wu et al. (2011) and Psofogiannakis and Froudakis (2009), showing that this process is rather endothermic.

In the present work, we study the viability of hydrogen spillover as a mechanism to hydrogen storage. For this purpose, we performed quantum mechanical calculations based on DFT, to determine the energy profiles of the aforementioned mechanistic steps. We used bent-C_54_H_18_ and planar-C_54_H_18_ structures as model systems for small and large diameter single-walled carbon nanotubes (SWCNT), respectively. Firstly, we calculate the interaction energy between H_2_ and the carbon substrate for the initial reaction on a finite CNT model H_2_ + C_54_H_18_ → H_2_@C_54_H_18_ without any catalyst. We found that to load the carbon structure with hydrogen from gas-phase is a rather endothermic process with reaction energies of 1.3–2.9 eV and 1.9–3.4 eV for small and large diameter SWCNTs, respectively. Furthermore, the barrier to H_2_ chemisorption is larger than 3.5 eV. On the other hand, the recombination of H atoms to form a H_2_ molecule, in the absence of any catalyst, requires overcoming an energy barrier of 0.4, 1.6 and 2.0 eV when the H pair is removed from the *meta, para* and *ortho* positions of large diameter SWCNTs, respectively. This barrier becomes higher [1.2 (*meta*), 2.9 (*ortho*) and 2.9 eV (*para*)] when H atoms are removed from the small diameter SWCNTs. However, the full dehydrogenation of CNT without catalyst support by heating up to 600°C has been reported (Nikitin et al., 2005).

To study the effect of the catalyst on loading and unloading of hydrogen, a Pt_4_ cluster was used as a model catalyst. In accordance with many reports (Zhou et al., 2007; Chen et al., 2009; Gomez et al., 2011), we found that H_2_ is split into H atoms spontaneously on the cluster, i.e., without any energy barrier. However, we found that the migration of H atoms from Pt_4_ to the carbon substrate defines the energetically most expensive step of the spillover mechanism with an energy barrier of about 2 eV which is 50% lower than that obtained for the non-catalyzed process. Furthermore, we have found relatively lower energy barriers for the Pt_4_ mobility on the substrate which is energetically favored over the diffusion of the H atoms (Chen et al., 2007a). Therefore, we propose that Pt_4_ mobility is the key to the hydrogenation of the SWCNT.

## Methods and computational details

**Figure 1:**
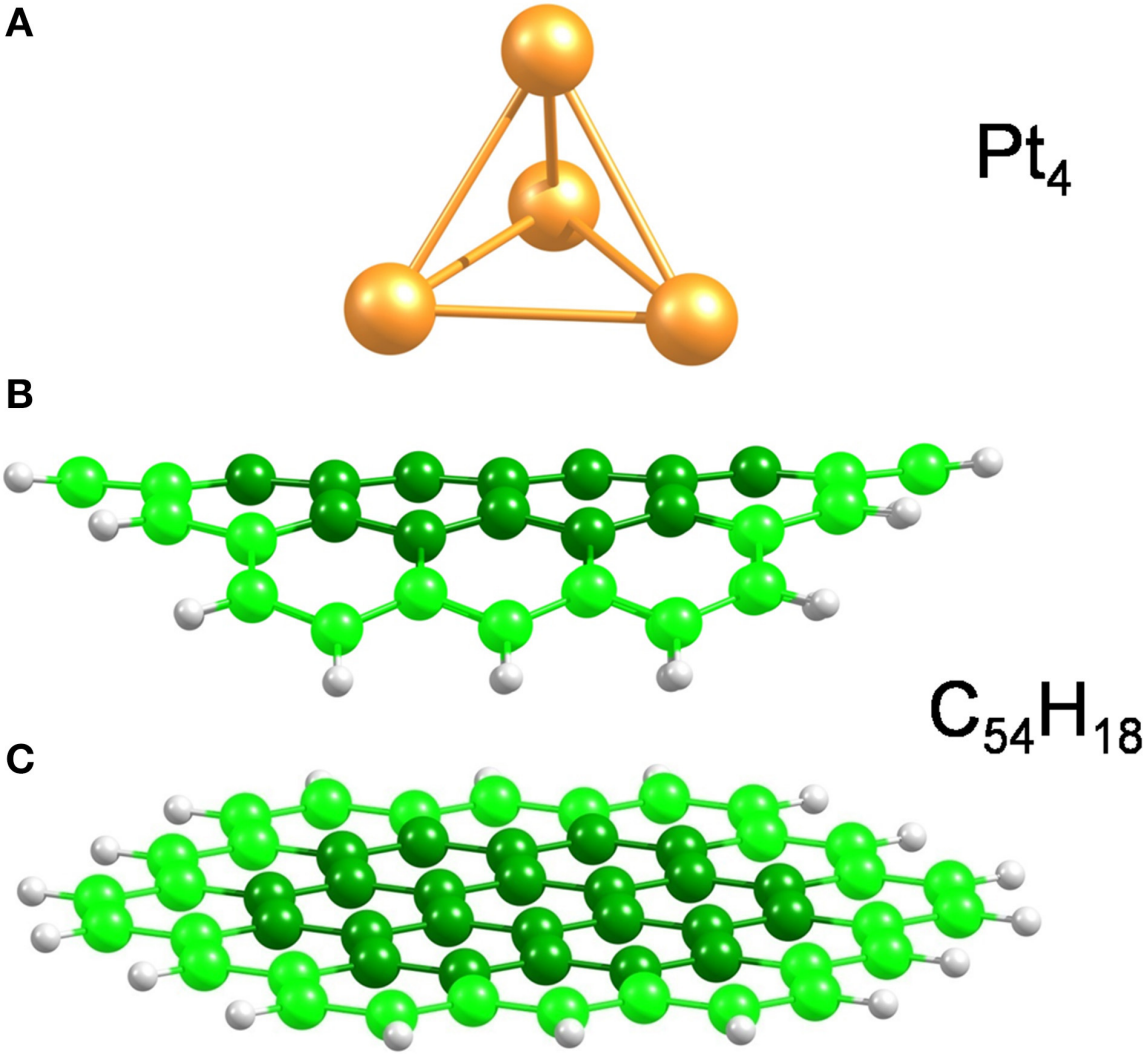
**Model structures used in the present work. (A)** Pt_4_ cluster, **(B,C)** bent and planar C_54_H_18_ structures to model small and large diameter SWCNTs, respectively. The bent carbon structure corresponds to the curvature of the (10,10) CNT. The carbon atoms at the edges are highlighted to stress that those, along with the H atoms, were kept fixed during structural optimizations.

Bent and planar circumcoronene (C_54_H_18_) structures were used to model the (10,10) CNT and a large diameter CNT or, equivalently, graphene (Figure 1), respectively. We have recently shown in the case of inorganic nanotubes that the properties of the large-diameter species are well-described by planar models (Ghorbani-Asl et al., 2013). Both models were simulated using the experimental bond lengths of 1.44 Å (White and Todorov, 1998; Saito et al., 2000) as initial geometry and with the outermost atoms fixed in the structural optimizations while the central atoms (darker color in Figure 1) were free to move. In earlier works (Jeloaica and Sidis, 1999; Mitchell et al., 2003a,b; Bonfanti et al., 2007; Psofogiannakis and Froudakis, 2009), a smaller coronene system has been shown to be a good model of the (0001) graphitic surface. A small platinum cluster, namely Pt_4_ (Figure 1), with tetrahedral symmetry, was used as a catalyst to dissociate the H_2_ molecule. Although the Pt_4_ cluster is much smaller than a real catalyst particle, it has been demonstrated that the H_2_ dissociation energy and the hydrogen desorption energy are independent of the platinum particle size (Zhou et al., 2007).

Note that from the electronic structure point of view, our circumcoronene model is a semiconducting material, while the (10,10) CNT is actually a metallic system. However, the electronics should not significantly influence the binding energies, as shown earlier by Yildirim et al. (2001).

According to our spin-dependent calculations, we found that the distorted tetrahedral Pt_4_ cluster in the triplet spin state is by 0.47 eV and 0.13 eV more stable than the singlet and quintet spin configurations, respectively. Therefore, all calculations were performed using the triplet state, in agreement with the work of Sebetci (2006). We have tested four different DFT functionals [see Table S1 in Supporting Information (SI)] in terms of the electronic structure of Pt clusters and its geometry. We have selected the PBE (Perdew et al., 1996) density functional as it is one of the least expensive functionals, it is often used in similar studies, and, most importantly, it correctly predicts the triplet configuration of the Pt system in its tetrahedral geometry.

All calculations have been carried out using density-functional theory (DFT) (Hohenberg and Kohn, 1964; Kohn and Sham, 1965), with the PBE (Perdew et al., 1996) exchange-correlation functional in combination with the def2-TZVP basis set as implemented in the Turbomole 6.5 software (Ahlrichs et al., 1989; Eichkorn et al., 1997). The employed basis set accounts for relativistic effects by using an effective core potential (ECP) for the 60 core electrons for Pt. The geometry optimization criteria for the forces and energy threshold were set to be smaller than 10^−4^ hartree per bohr and 10^−6^ hartree, respectively. The convergence criterion for the energy calculation during the self-consistent-field procedure was set to 10^−7^ hartree. We have used the m4 grid in all simulations.

The coordinate-driven interpolation method of finding an estimate for the barrier, which we have used here, can be considered equivalent to the nudged elastic band technique (all other degrees of freedom are fully relaxed at each step) where the coordinate of interest would be the C-H bond length.

To analyze the whole spillover process, we divided the study into several steps. Firstly, we calculated the energy barriers for loading and unloading the carbon substrate with H_2_ in absence of any catalyst, for our small cluster models. Activation (*E*^‡^) and reaction (Δ*E*) energies (also called here adsorption or chemisorption energies) were calculated for two H atoms disposed at the center of the coronene structure in *ortho, meta* and *para* configurations. To calculate *E*^‡^ the energy was scanned over the C-H distance (*d*_C−H_) starting from the optimized structures (*d*_C−H_ ~1.1 Å) up to the point at which the H_2_ molecule was already formed (*d*_*C*−*H*_ ~3.6 Å) (see Movie 00 in SI). Δ*E* was calculated with reference to H_2_ at infinite distance from the C_54_H_18_ substrate. In these optimizations the carbon and hydrogen atoms at the edges (i.e., the 30 outer C atoms and the 18 H atoms) in C_54_H_18_ were kept fixed, while the rest of the structure was relaxed (see Figure 1).

In the second step, we have analyzed the dissociation of H_2_ on the Pt_4_ cluster omitting the influence of the carbon substrate. The calculations were performed by scanning over the Pt_4_–H_2_ distances starting from the optimized Pt_4_ and H_2_ molecules, separated by 2.5 Å and going down to 1.5 Å, where the hydrogen molecule was dissociated and H atoms were attached to the platinum cluster (See Figure S1 in SI). The only constraint imposed in this simulation was the Pt-H distance.

Next, we have studied the migration of hydrogen atoms to and from the hydrogenated catalyst (Pt_4_H_2x_ with x = 1 − 6) to the carbon substrates (for an example, see Movie 01 in the SI). As before, we imposed constraints on the edges of the carbon models (see Figure 1) while the 24 carbon atoms at the center of the circumcoronene structure and the Pt_4_H_2x_ cluster were allowed to move.

Finally, we have investigated the mobility of the Pt_4_ cluster on the carbon substrates. We have taken into account the influence of the Pt_4_ cluster loading with hydrogen where we have compared bare and fully saturated clusters as limiting cases, *e.i* C_54_H_18_–Pt_4_ and C_54_H_18_–Pt_4_H_12_, respectively. The tetrahedral Pt_4_ and Pt_4_H_12_ clusters were pre-optimized on top of C_54_H_18_ substrate using different starting points. During these optimizations the edges of the C_54_H_18_ structure were kept fixed, while the Pt_4_ and Pt_4_H_12_ clusters and the central carbon atoms were allowed to move. All the different starting points studied here resulted in only three different configurations; bare Pt_4_ cluster with tip down (end on), tip up (face on) and saturated Pt_4_H_12_ cluster with edge on. Afterwards, for the resulting structures, the platinum clusters were shifted along the carbon substrate with a step size of 0.5 Å (see Movie 02 in the SI) and rotated parallel to the substrate in steps of 2° (see Movie 03 in the SI). We have kept the C-Pt distance fixed during the scanning to estimate the influence of the substrate's curvature on the mobility of the catalyst.

## Results

### Hydrogen chemisorption and recombination in absence of catalyst

**Figure 2:**
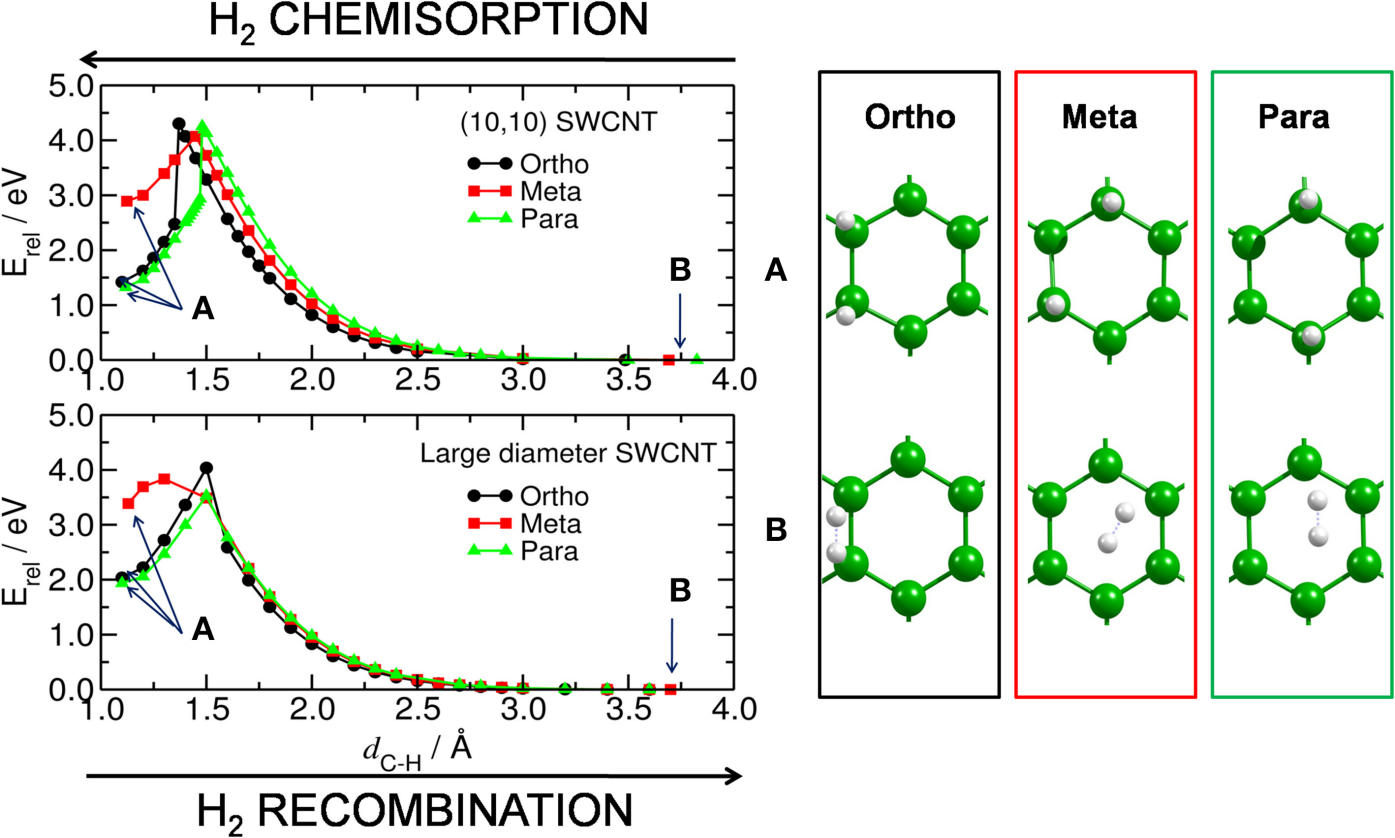
**H_2_ chemisorption/recombination on carbon substrate**. (Left) energy barriers for the chemisorption/recombination (*E*^‡,*s*^/*E*^‡,*d*^) of two H atoms on the bent (upper) and planar (lower) C_54_H_18_ substrates, corresponding to the (10,10) and large diameter SWCNT models, respectively. Blue arrows inside the plot point to the equilibrium energy for **(A)** the structure with two H atoms attached to the substrate (i.e., H_2_@C_54_H_18_) and, **(B)** for the H*_2_* detached from C_54_H_18_ and located at a distance of ~3.6 Å from the substrate. (Right) corresponding central part of the equilibrated structures for H_2_@C_54_H_18_ and H_2_ + C_54_H_18_.

The energy required to hydrogenate the carbon substrate in the absence of any catalyst was calculated for the bent and planar models according to the reaction H_2_ + C_54_H_18_ → H_2_@C_54_H_18_(see Table 1 and Figure 2). The results show that the process is rather endothermic, with Δ*E* of 2.0, 3.4, and 1.9 eV for two H atoms attached to the large diameter CNT model in *ortho, meta*, and *para* positions, respectively. The corresponding activation energies to chemisorb (*E*^‡,s^) the two H atoms are 4.0 (*ortho*), 3.8 (*meta*) and 3.5 eV (*para*). For the curved (10,10) CNT, the endothermicity is reduced by ~0.6 eV to 1.4, 2.9 and 1.3 eV for the two H atoms attached in *ortho, meta* and *para* positions, respectively. This is in accordance with previous studies on the influence of curvature on the adsorption energy in CNTs, which showed strong reduction with increased curvature (Gao et al., 2011). The *E*^‡,s^ is oppositely affected by the tube curvature, increasing by 0.2–0.7 eV. According to our calculations, *ortho* and *para* are the preferred adsorption positions for hydrogen, which is in agreement with earlier works (Andree et al., 2006; Hornekær et al., 2006b). Becher et al. (2003) concluded that reversible hydrogen storage at ambient temperature is unlikely based on the endothermic adsorption energies, which range from 2–3 eV. It should be noted, however, that the adsorption energy per hydrogen atom of nearly fully hydrogenated structures is different compared to the initial step (initial hydrogenation of the substrate); depending on the curvature, these values can get much smaller (Gao et al., 2011).

**Table 1:** **Reaction (Δ*E*) and activation (*E*^‡^,**s**) energies (eV) for the sorption of two H atoms on the planar and bent C_54_H_18_ substrates disposed at *ortho, meta*, and *para* positions**.

	**Ortho**			**Meta**			**Para**		
	**ΔE**	**E^‡,s^**	**E^‡,d^**	**ΔE**	**E^‡,s^**	**E^‡,d^**	**ΔE**	**E^‡,s^**	**E^‡,d^**
Planar-C_54_H_18_	2.0	4.0	2.0	3.4	3.8	0.4	1.9	3.5	1.6
Bent-C_54_H_18_	1.4	4.3	2.9	2.9	4.0	1.2	1.3	4.2	2.9

E^‡,d^ *stands for the activation energy to recombine two H atoms (desorption) out of the *ortho, meta* and *para* configurations*.

The discontinuities in the computed potential energy curves are not due to an inappropriate reaction coordinate, but rather due to hysteresis effects in the applied DFT technique when treating a curve crossing. Forming the two C-H bonds from closed-shell H_2_ and the coronene requires a spin-uncoupling of both systems to reach the bonding singlet state formed from triplet H_2_ and triplet coronene (Triguero et al., 1998). This internal rehybridization of both systems simultaneously may be difficult to achieve and the computed curves erroneously follow the repulsive state beyond the crossing point before collapsing to the correct lower state. However, since even the energy barrier estimated from the lower state is too high for the reaction to be realistic we take this lower value as estimate of the barrier.

Furthermore, Hornekær et al. (2006a) reported, using scanning tunneling microscopy and DFT calculations, that recombination of two H atoms on graphite occurs only from the *para* position with a barrier of 1.4 eV. This is in close agreement with our *E*^‡,*d*^ from the *para* position of 1.6 eV for the planar model of a large diameter CNT. We obtained an *E*^‡,*d*^ of 2.9 eV for the (10,10) CNT. The *E*^‡,*d*^ from the *ortho* position was found to be 2.0 and 2.9 eV for the planar and bent models, respectively. These results indicate that, without catalyst, the chemisorption and recombination of hydrogen on the carbon materials would require large energies to overcome the barriers. Considering finally the least favorable adsorption site, the *meta* position, we find low values of 1.2 and 0.4 eV for the recombination on the curved and planar substrates, respectively. This is not surprising since the *meta* position is the least favored one.

### Catalyzed hydrogen chemisorption and recombination on carbon substrates

**Figure 3:**
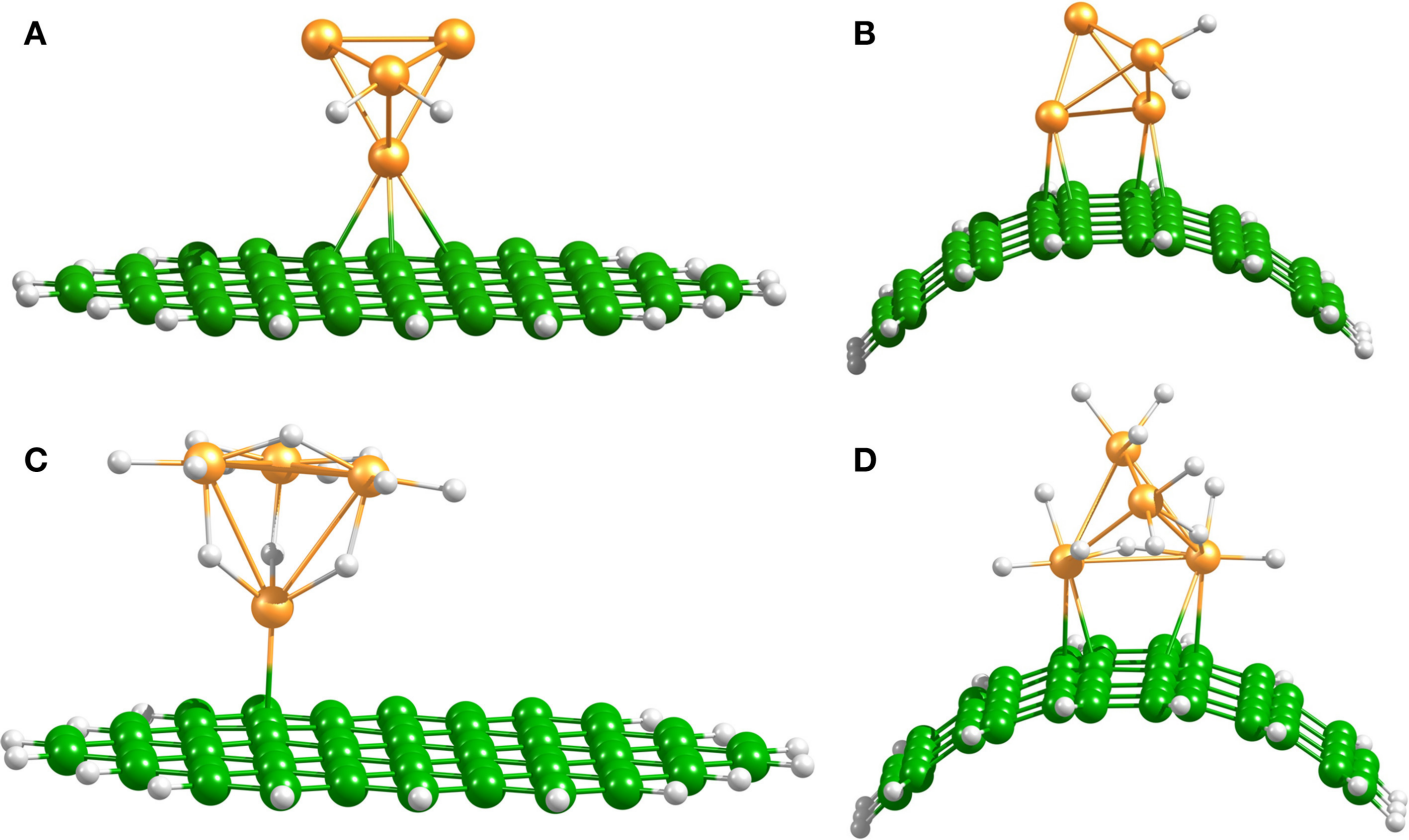
**Spillover models**. Optimized Pt_4_–2H and Pt_4_–12H structures on planar **(A,C)**, and bent **(B,D)** C_54_H_18_ structures.

Platinum nanoparticles are commonly used as transition-metal catalysts to hydrate surfaces via the spillover process (Somorjai, 1994; Lueking and Yang, 2004). In agreement with previous reports (Zhou et al., 2007; Chen et al., 2009; Gomez et al., 2011) we found that the dissociation of H_2_ on Pt_4_ occurs without any energy barrier and up to 6 H_2_ molecules can be dissociated and accommodated on this catalyst. Figure 3 shows the optimized structures of the hydrogenated Pt_4_ cluster attached to the bent and planar carbon substrates. It is important to mention that the fully hydrogenated Pt_4_ cluster retains its tetrahedral shape, but an increase of the Pt–Pt distance of maximum 0.3–0.5 Å is generated due to saturation. Chen et al. have reported an increase of the Pt–Pt bond lengths of 0.01–0.3 Å for a Pt_6_ cluster at high H coverage (Chen et al., 2007b).

**Figure 4:**
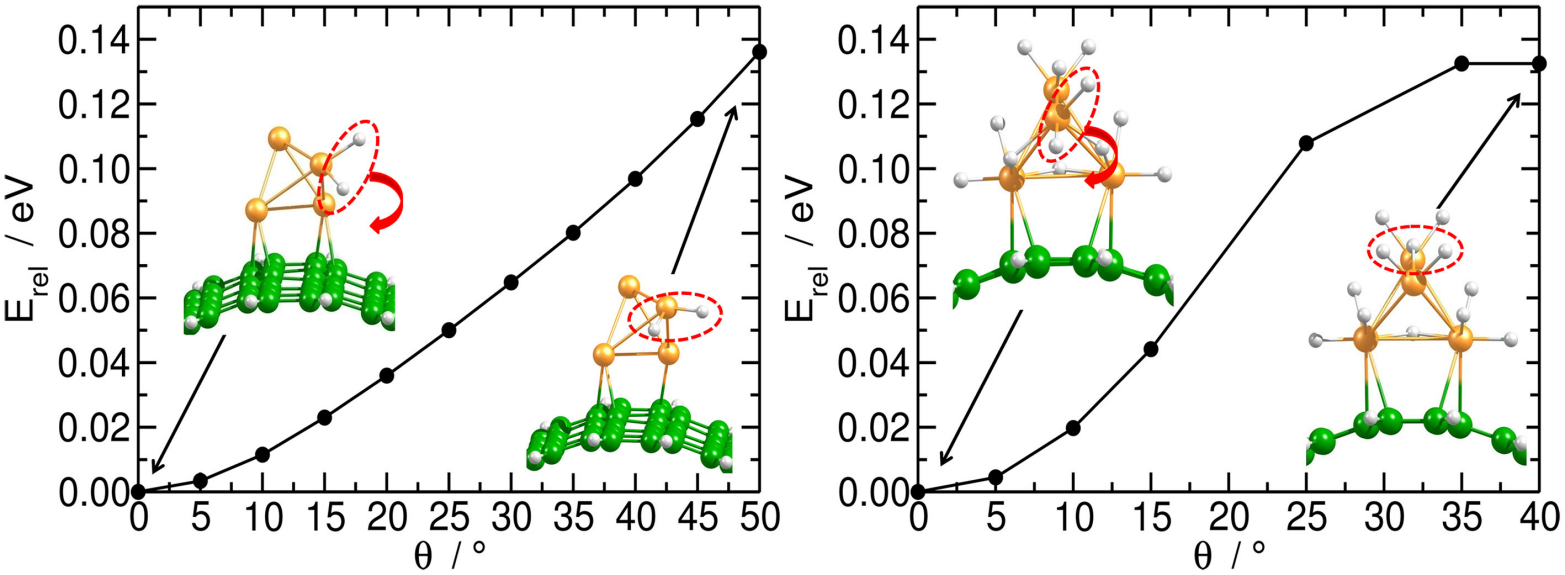
**Rotation of H atoms on Pt_4_ catalyst**. Energy profile for the rotation of two H atoms on bi-hydrogenated (left) and dodeca-hydrogenated (right) clusters on bent C_54_H_18_ structure. The energy is related to the equilibrium geometry.

Independent of the number of H atoms attached to the catalyst, the Pt_4_ cluster binds to the planar substrate with one Pt atom close to the C atoms and with the H atoms oriented nearly parallel to the carbon surface. The binding energies for the bare and fully saturated Pt_4_ cluster to the planar substrate are strongly exothermic with values of −2.25 and −1.50 eV, respectively. Surprisingly, we find that the presence of the Pt_4_ cluster on the substrate does not affect the structure of the substrate. On the bent substrate we find a Pt_4_ structure with two Pt atoms bonded to the CNT. The binding energies for the bare and fully saturated Pt_4_ with the bent C_54_H_18_ are in this case −2.81 and −2.04 eV, respectively. Moreover, on the bent substrate the H atoms are oriented nearly perpendicular to the carbon surface. For the subsequent migration of two H atoms to the carbon substrate, it is required to rotate the H atoms to face the carbon substrate in a parallel manner (Figure 4). The rotation barrier of around 0.14 eV was calculated using the same constraints as shown in Figure 1, but additionally a dihedral angle was fixed to avoid the rotation of the H atoms in each point of the energy scan.

**Figure 5:**
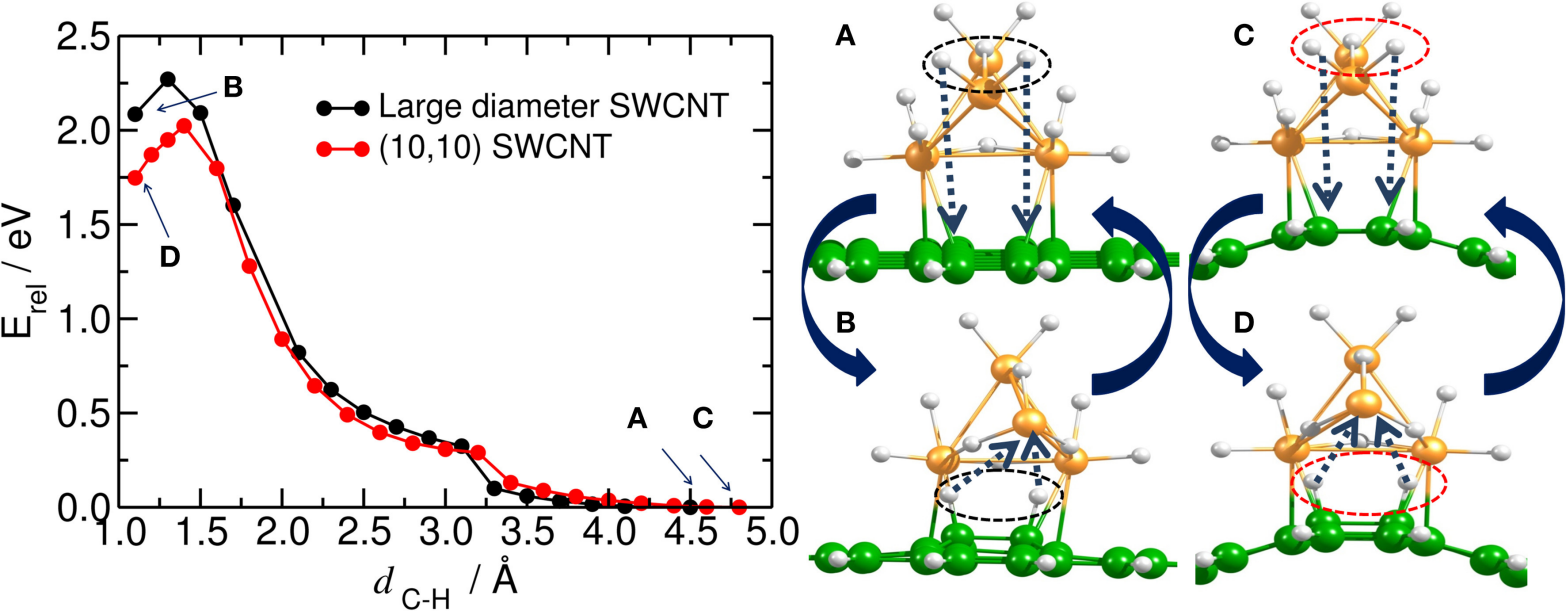
**Hydrogen spillover on carbon substrate**. (Left) Energy profile for the simultaneous migration of the first two H atoms from the dodeca-hydrogenated Pt_4_ cluster to the bare carbon substrate (i.e., initial hydrogenation). Equilibrium energy for the structures in which all H atoms are initially attached to the platinum cluster and the carbon substrate is bare are pointed out by blue arrows as **(A,C)**, and shown in the right upper panel. **(B, D)** inside the plot (and in the right lower panel) stand for the equilibrium energy (and structures) in which the first two H atoms are chemisorbed by the carbon substrate. This plot can also be read as the migration of the H atoms from the carbon substrate to the deca-hydrogenated Pt_4_ cluster.

The activation barrier for one H atom to migrate from the Pt_4_ cluster to carbon has been calculated by Wu et al. (2011) and by Psofogiannakis and Froudakis (2009) using DFT. The authors found that the estimated barrier for migration of one H atom from the fully saturated Pt_4_ cluster to the carbon substrate is 2.7 eV (Wu et al., 2011) and 2.6 eV, (Psofogiannakis and Froudakis, 2009) respectively. Our results show that the barrier for the simultaneous migration of two H atoms from the dodeca-hydrogenated Pt_4_ cluster to the bent and planar surface is 2.0 eV and 2.3 eV, respectively (see Figure 5 and the Movie 01 in SI). On the other hand, we have observed that when bending the saturated Pt_4_ cluster toward the substrate, the carbon structure is puckered down. This additionally supports the fact that the hydrogen migration from the catalyst to the carbon is not a straightforward process and even a rotational motion of the catalyst cluster does not lower the energy barrier (see Figure S2 in SI).

Wu et al. (2011) have shown that for pure graphene, in which an H atom was manually put on a C atom, the hydrogen migrates back to the Pt_4_ cluster after structural relaxation. In our calculations, we observed that this effect depends on the degree of saturation of the Pt_4_ cluster and the substrate itself. From simple optimization calculations, we observed that the Pt_4_ cluster with eight hydrogen atoms attached (Pt_4_H_8_) spontaneously collects two out of four H atoms that were located manually on the substrate in one hexagon ring, but it does not collect any when only one pair of H atoms is attached to the substrate in *ortho* position (see Movie 04 and Movie 05 in SI). Similarly, we observed that the bare Pt_4_ cluster spontaneously collects H atoms from the graphitic surface, in which two hydrogen atoms were manually attached (see Movie 06 in SI).

To investigate the role of the substrate and catalyst saturation, we calculated the energy profile for subsequent hydrogen migrations; a second pair of H atoms migrating from the dodeca-hydrogenated (Pt_4_H_12_) cluster to the (10,10) CNT surface modified such that there were two H atoms initially attached to the substrate. These two hydrogens were disposed into three different positions of the same hexagonal ring (Figure S3 in SI). From these calculations we found that the energy barrier for the subsequent migration remains essentially unchanged (~2.0 eV), while the adsorption energy for the subsequent hydrogenations decreases by around 0.5 and 0.1 eV depending on the relative arrangement of the first two hydrogens and the second pair (see Figure S3 in SI). This means that the adsorption of H atoms becomes less endothermic.

To investigate the effect of the cluster saturation we also performed similar calculations for the migration of the first two hydrogens from a deca-hydrogenated (Pt_4_H_10_) cluster to the bare C_54_H_18_ surface. However, we did not observe any significant change in the energetics. In conclusion, the energy barrier for the initial hydrogenation of the substrate is around 2 eV and is essentially unaffected by the level of pre-hydrogenation of the substrate. Nevertheless, the presence of the H pair on the substrate lowers the hydrogen adsorption energy up to 0.5 eV, lowering the level of the endothermic process.

### Platinum cluster mobility on carbon nanotubes

**Figure 6:**
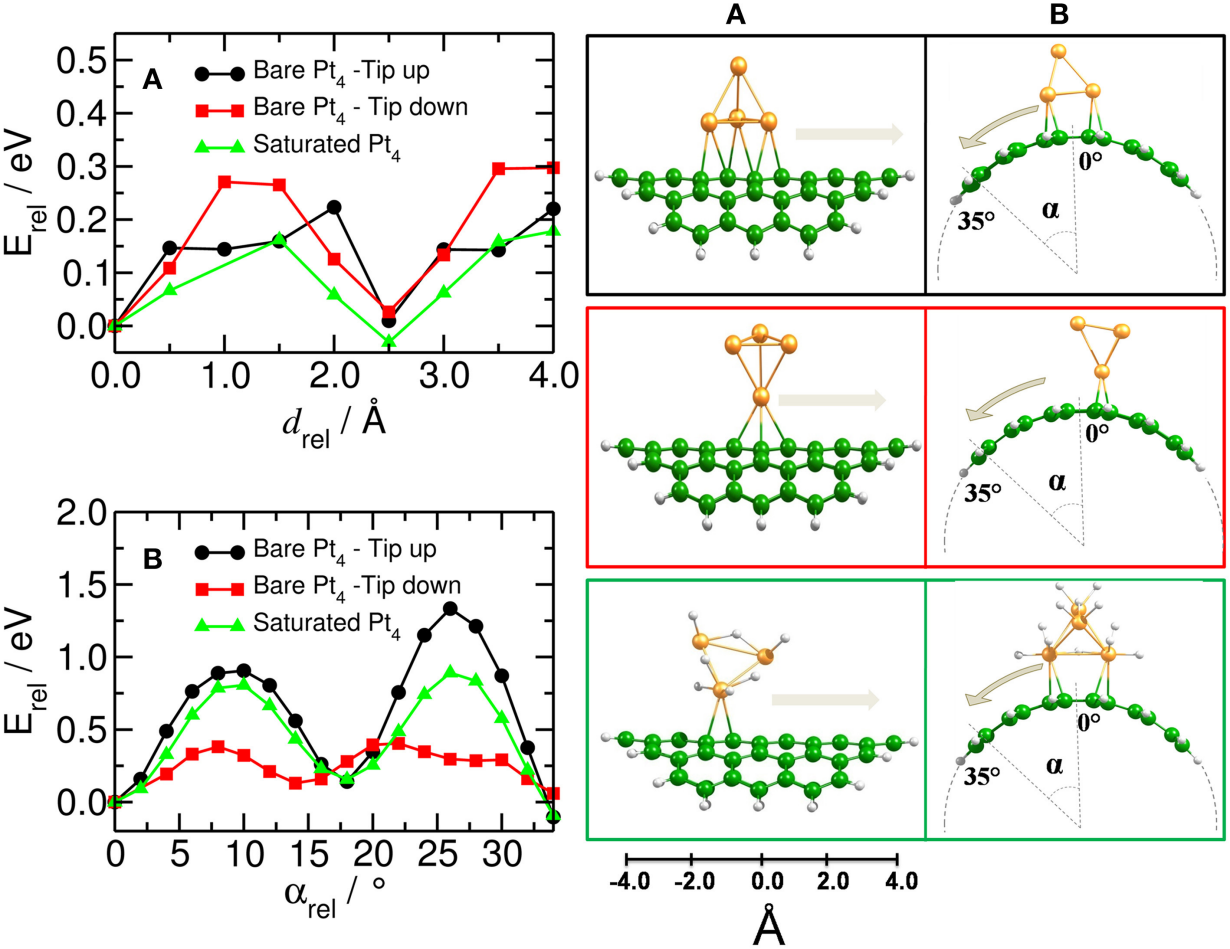
**Mobility of catalyst on bent carbon substrate**. (Left) Energy profile for moving the Pt_4_ cluster **(A)** along and **(B)** around the curved C_54_H_18_ structure. (Right) Corresponding equilibrium positions of the Pt_4_ clusters with tip up (black) and down (red) and for the fully saturated Pt_4_ cluster (green). Energy is related to the respective initial optimized structures.

Figure 6 shows the relative energy for the migration of the platinum catalyst along and around the (10,10) CNT. All energies are related to the optimized C_54_H_18_–Pt_4_ and C_54_H_18_–Pt_4_H_12_ structures. We have found that the Pt_4_ cluster moves along the CNT axis more easily than around the tube. The largest barriers for the Pt_4_ mobility along the CNT axis were calculated for the case when the cluster binds to the substrate with the lowest coordination number (i.e., that is tip-down), resulting in an activation barrier of about 0.30 eV. This barrier is further reduced for the Pt_4_ cluster with tip-up or the saturated cluster for which we find barriers of 0.22 eV and 0.16 eV, respectively (see Figure 6A). Thus, platinum clusters move more easily on the carbon substrate at the full saturation limit.

In order to move the saturated Pt_4_ cluster around the tube, it is required to overcome a barrier five times higher than when moving it along the tube (0.89 eV against 0.16 eV, *cf*. green lines in Figures 6A,B), suggesting that the curvature of the surface has an important effect on the Pt_4_ mobility. This was also suggested by Chen et al. (2007a).

**Figure 7:**
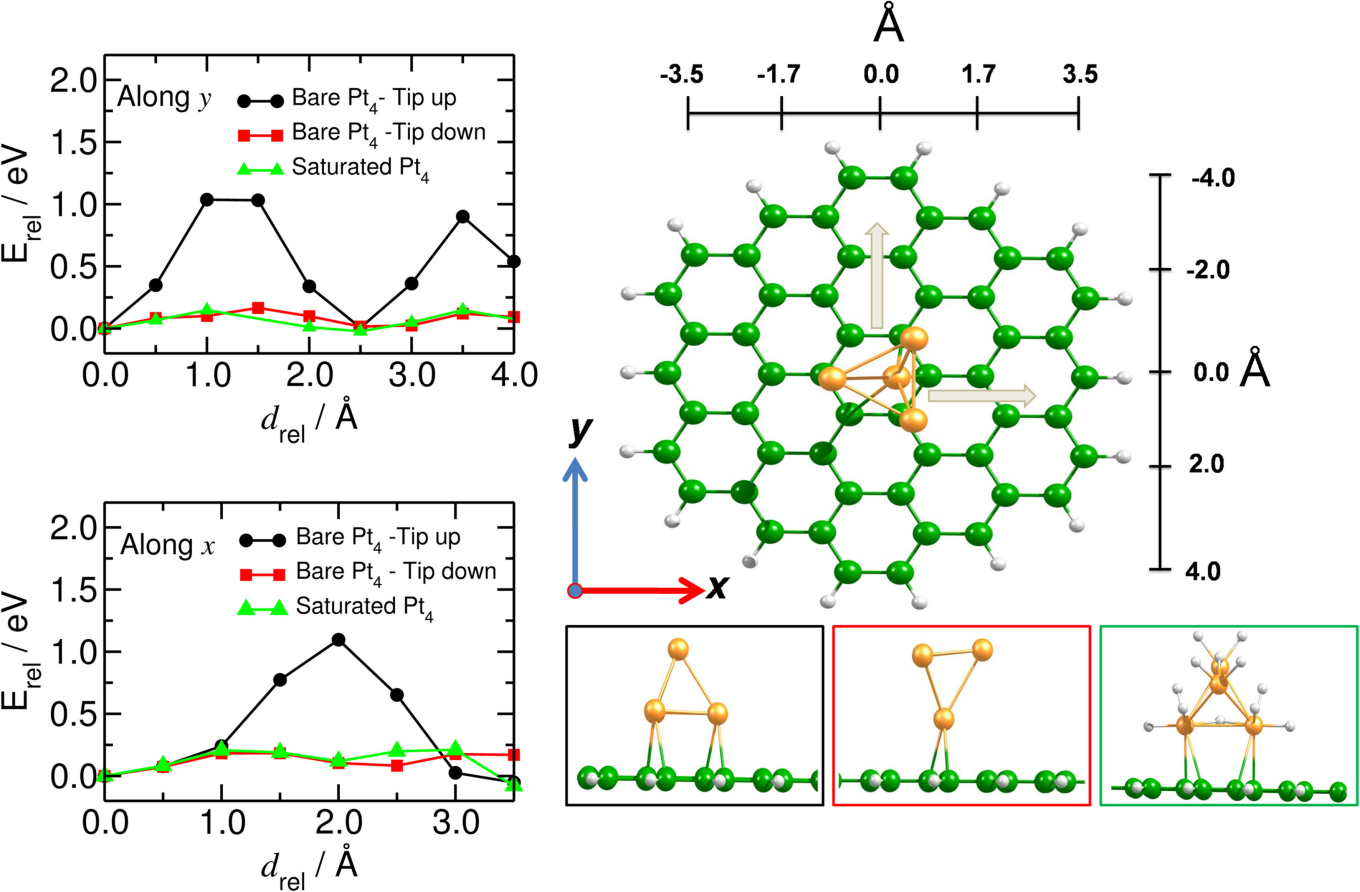
**Mobility of catalyst on planar carbon substrate**. (Left) Energy profile of the mobility of the Pt_4_ cluster (along, *y*, and across, *x*) on the flat C_54_H_18_ structure. (Right) Corresponding equilibrium positions of the Pt_4_ cluster with tip up (black), tip down (red) and for the fully saturated Pt_4_ cluster (green). *x* and *y* indicate the directions of the Pt_4_ motion.

We have analyzed the mobility of the Pt_4_ cluster also on the flat C_54_H_18_ structure, to model the situation of large diameter carbon nanotubes (Figure 7). We found that the energy barrier does not depend on the direction of the cluster motion (zigzag or armchair), but it does depend on the C-Pt coordination number. Thus, the highest barrier for the Pt_4_ mobility (of about 1.0 eV) was found for the situation where the cluster binds to more than three carbon atoms at an average C-Pt distance of 2.28 Å and a binding energy of −2.25 eV. Similar to the bent model, the saturated cluster with binding energy of −1.50 eV moves easily along the carbon surface with barrier of only 0.2 eV. These results show that the migration of the catalyst is preferable for the saturated catalyst and it is strongly influenced by the curvature of the tube.

## Conclusions

We have investigated the individual steps of hydrogen storage by the spillover mechanism and complemented our calculations with data from the literature. First, on carbon nanostructures, such as small-diameter SWCNT, large-diameter SWCNT and graphene, hydrogen can chemisorb and desorb only by overcoming a significant energy barrier. This suggests that those structures can store hydrogen for a long time at very high gravimetric and volumetric capacities. Hydrogen is covalently bound, and the endothermic adsorption energies are between 1.3 and 2.9 eV for the small diameter SWCNT and between 1.9 and 3.4 eV for the large diameter SWCNT. For smaller SWCNTs, even lower energy values are reported in the literature (Gao et al., 2011). The work of Chen et al. (2007a) reports a very low hydrogen mobility on the carbon surface with a barrier of 1.42, 1.09 and 0.78 eV associated to its motion on (5,5), (9,9) CNTs and graphene, respectively. Thus, a traditional spillover mechanism where hydrogen atoms migrate quickly along the surface is not possible for graphitic structures.

Loading and unloading these structures with hydrogen without a catalyst would require overcoming barriers of more than 3.5 eV and 1.5 eV to chemisorb and to remove, respectively, a pair of H atoms from *ortho* and *para* positions. However, the barrier for loading the carbon substrate can be significantly reduced by using a catalyst. Moreover, the sorption energy becomes less endothermic for smaller diameter SWCNTs.

The most promising catalysts for hydrogen activation are platinum nanoparticles. These particles spontaneously dissociate H_2_ from the gas phase and can take up a large number of hydrogen molecules. For example, our Pt_4_ model cluster is saturated after adsorbing six H_2_ molecules. Bare and hydrogenated Pt clusters bind to the *sp*^2^ carbon surface with binding energies of at least −2.25 eV and −1.50 eV, respectively. The catalyst lowers the barriers for the initial surface hydrogenation by about 50%. In addition, we found that the presence of a pair of H atoms on the substrate does not affect the activation barrier for subsequent hydrogenation, but it reduces the endothermic adsorption energy for the next H pair by about 0.5 eV.

We have further established that Pt nanoparticles are very mobile on the *sp*^2^ carbon substrates and can relocate nearly barrier-free. Thus, instead of the traditional spillover mechanism from a static catalyst particle followed by hydrogen diffusion along the support, it is more likely that the Pt nanoparticles move over the substrate surface and thus load/unload H_2_ locally. The high mobility of the Pt nanoparticles has, however, a crucial drawback: due to their high mobility we expect the particles to cluster to larger Pt aggregates, which ultimately terminates the efficiency of the catalyst and the working principle of a hydrogen storage device based on this concept.

### Conflict of interest statement

The authors declare that the research was conducted in the absence of any commercial or financial relationships that could be construed as a potential conflict of interest.
