# Supplementary material for: Theoretical analysis of hydrogen spillover mechanism on carbon nanotubes
Source: Front Chem. 2015 Feb 2;3:2. doi: 10.3389/fchem.2015.00002 (PMC4313777; doi:10.3389/fchem.2015.00002)
Supplement: Supplementary file 1 [file Table1.DOCX]

***Supplementary Material***

**Theoretical analysis of hydrogen spillover mechanism on carbon nanotubes**

**Rosalba Juarez-Mosqueda^1^, Andreas Mavrandonakis ^1^, Agnieszka Kuc ^1^, Lars G. M. Pettersson^2^, Thomas Heine^1*^**

^1^ School of Engineering and Science, Jacobs University Bremen, 28759 Bremen, Germany

^2^Department of Physics, AlbaNova University Center, Stockholm University, S-106 91 Stockholm, Sweden

*** Correspondence:** School of Engineering and Science, Jacobs University Bremen, 28759 Bremen, Germany. E-mail: T.heine@jacobs-university.de

1. **Supplementary Data**

**Movie 00**: Hydrogen recombination from the planar C_54_H_18_ carbon substrate in absence of the catalyst. See the corresponding energy profile in **Figure 2**.

**Movie 01**: Simultaneous migration of the first two H atoms from the dodeca-hydrogenated Pt_4_ cluster to the bare bent carbon substrate. See the red line in **Figure 5** for the corresponding energy profile.

**Movie 02:** Mobility of the bare Pt_4_ cluster along the bent C_54_H_18_ substrate. See the corresponding energy profile in **Figure 6 A**.

**Movie 03:** Mobility of the bare Pt_4_ cluster around the bent C_54_H_18_ substrate. See the corresponding energy profile in **Figure 6 B**.

**Movie 04**: Spontaneous migration of two H atoms from the tetra-hydrogenated bent carbon substrate (C_54_H_18_-4H) to the octa-hydrogenated Pt_4_H_8_ cluster.

**Movie 05**: Geometry optimization of the octa-hydrogenated Pt_4_H_8_ cluster on top of the bi-saturated bent carbon substrate (C_54_H_18_-2H). No spontaneous migration of H atoms is observed.

**Movie 06**: Spontaneous migration of two H atoms from the bi-saturated planar carbon substrate (C_54_H_18_-2H) to the bare Pt_4_ cluster.

1. **Supplementary Figures and tables**


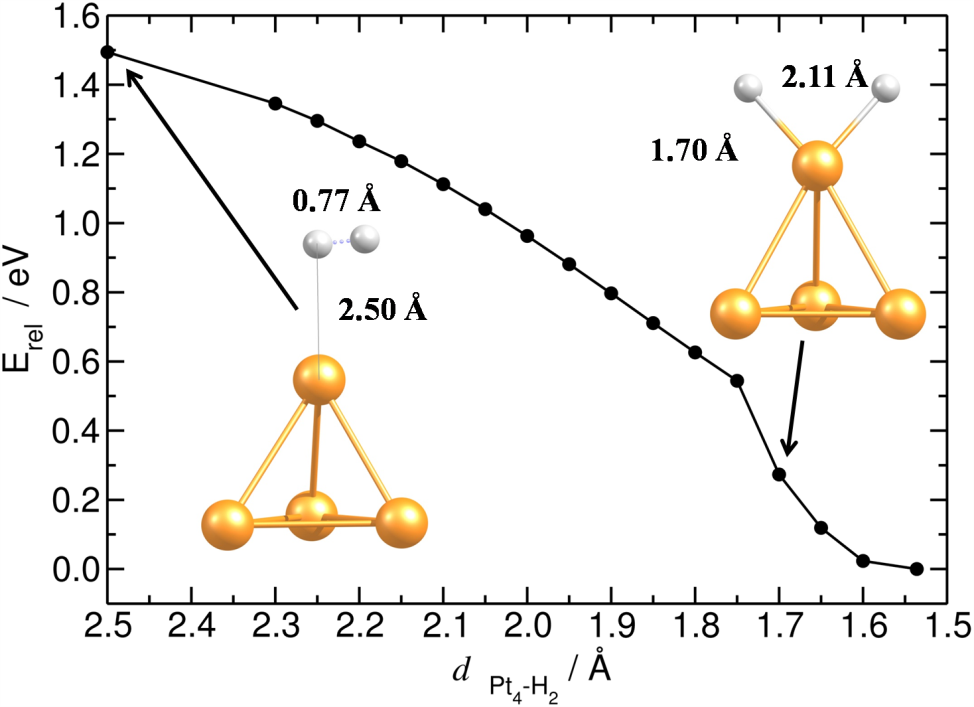


**Figure S1**. **Catalyzed hydrogen splitting**. Energy-free H_2_ molecule splitting on the Pt_4_ cluster.


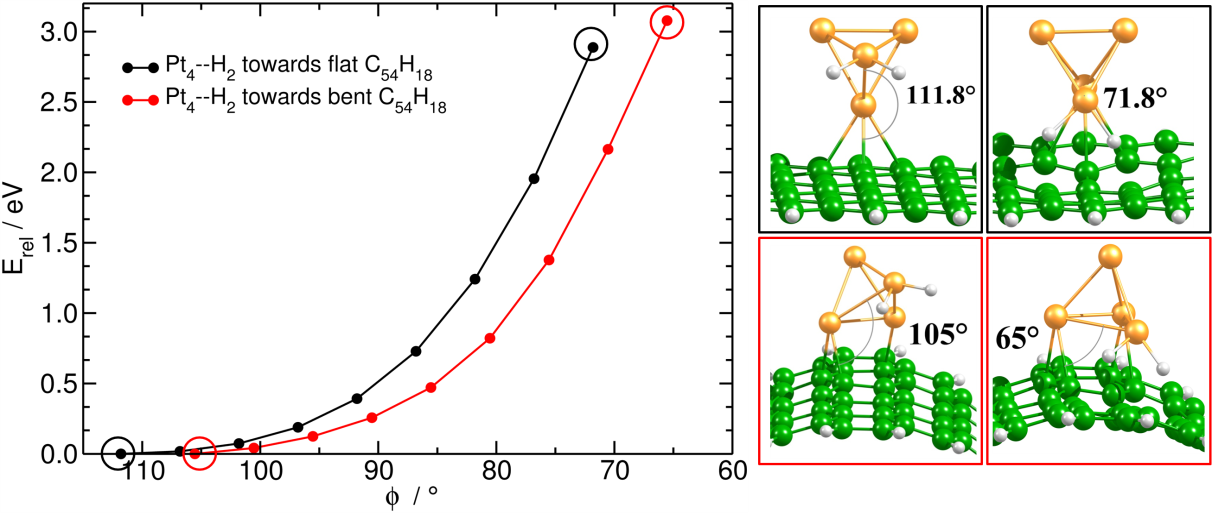


**Figure S2**. **Migration of two H atoms by bending Pt_4_H_2_ cluster**. Energy profile of bending the Pt_4_-2H cluster towards the surface of the planar (black) and bent (red) C_54_H_18_. Energy is related to the equilibrium systems shown in the first column of the right panel for planar (black) and bent (red) substrates. ϕ indicates the Pt-Pt-C angle.

**
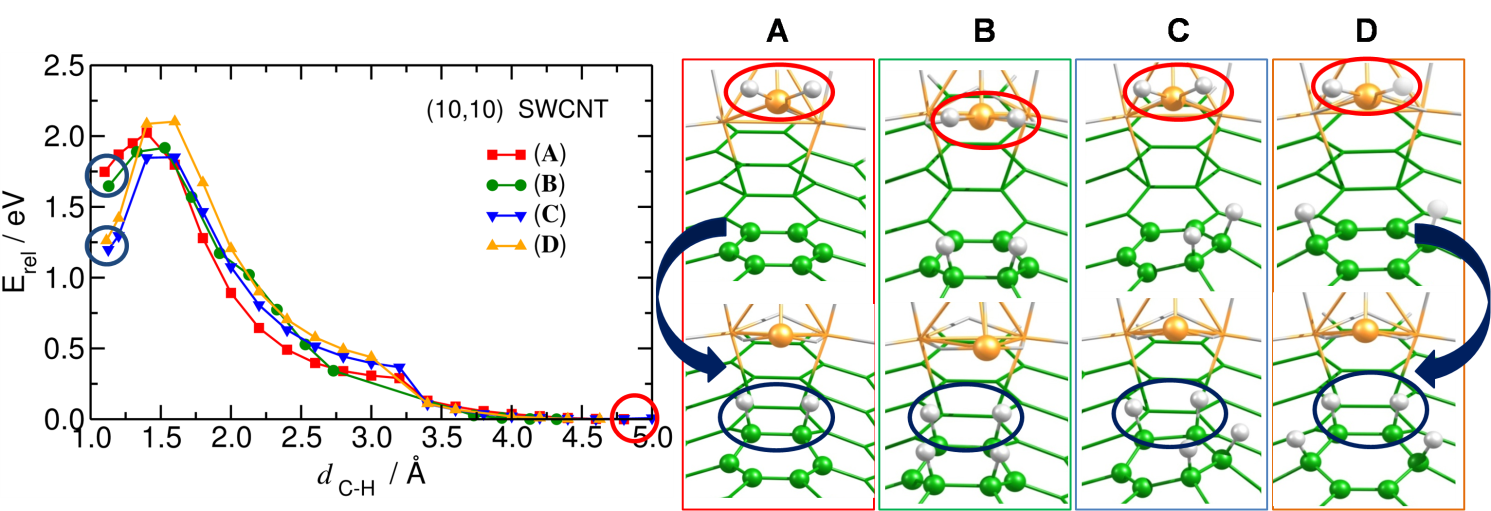
**

**Figure S3**. **Hydrogen spillover on bent carbon substrate – influence of hydrogen saturation.** (**A**) Energy profile for the initial migration of the first two H atoms from the dodeca-hydrogenated Pt_4_H_12_ cluster to the non-hydrogenated C_54_H_18_ substrate. The energy profile for the subsequent migration of a second pair of H atoms from the Pt_4_H_12_ cluster to the bi-hydrogenated C_54_H_18_-2H substrate is shown in (**B**) green, (**C**) blue and (**D**) orange. The corresponding equilibrated structures are shown in the right panels indicating the different arrangements of H atoms in the substrate. Upper right panels show the case in which the two migrant atoms are still attached to the Pt cluster (*d*_C-H_ ~ 5.0 Å), while the lower panels displayed the equilibrated structures in which the migrated H atoms are chemisorbed to the substrate (*d*_C-H_ ~ 1.1 Å). Equilibrated structures are also indicated in the plot with red and blue circles.

**Table S1.** **Electronic spin-state stability of tetrahedral Pt_4_ cluster calculated with four different DFT functionals.** Relative energies (in eV) of singlet and quintet configurations with respect to the most stable triplet spin state of Pt_4_ cluster.

| **Spin configuration** | **PBE** | **PBE0** | **B3LYP** | **BLYP** |
| --- | --- | --- | --- | --- |
| Singlet | 0.47 | 0.15 | 0.15* | 0.44 |
| Triplet | 0.00 | 0.00 | 0.00 | 0.00 |
| Quintet | 0.13 | 0.18 | 0.10 | 0.15 |

*The Pt_4_ cluster loses its tetrahedral shape.
